# Scalar field as a perfect fluid: thermodynamics of minimally coupled scalars and Einstein frame scalar-tensor gravity

**DOI:** 10.1140/epjc/s10052-023-11186-7

**Published:** 2023-01-16

**Authors:** Valerio Faraoni, Serena Giardino, Andrea Giusti, Robert Vanderwee

**Affiliations:** 1 Department of Physics and Astronomy, Bishop’s University, 2600 College Street, Sherbrooke, QC J1M 1Z7 Canada; 2 Max Planck Institute for Gravitational Physics (Albert Einstein Institute), Callinstraße 38, 30167 Hannover, Germany; 3 Institute for Theoretical Physics, Heidelberg University, Philosophenweg 16, 69120 Heidelberg, Germany; 4 Institute for Theoretical Physics, ETH Zurich, Wolfgang-Pauli-Strasse 27, 8093 Zurich, Switzerland

## Abstract

We revisit the analogy between a minimally coupled scalar field in general relativity and a perfect fluid, correcting previous identifications of effective temperature and chemical potential. This provides a useful complementary picture for the first-order thermodynamics of scalar-tensor gravity, paving the way for the Einstein frame formulation (which eluded previous attempts) and raises interesting questions to further develop the analogy.

## Introduction

Minimally coupled scalar fields are ubiquitous in theoretical physics and especially in cosmology. One of the simplest examples is quintessence, a proposal of dynamical dark energy represented by a scalar field, that aims to avoid the fine-tuning issues of a cosmological constant and could explain the current accelerated expansion of the universe in the context of general relativity (GR) [1, 2]. Also more elaborated scalar field models with non-canonical kinetic term are considered for dark energy, e.g., in the so-called k-essence [3–5]. Other exotic models of scalar-field based quintessence include, e.g., the string-inspired tachyon condensate [6–10].

The equivalence between a scalar field minimally coupled to the curvature endowed with timelike gradient and a perfect fluid is now well-established and has been the subject of a substantial literature ([11–18] and references therein). The generalization of this equivalence to imperfect fluids has proven to be even more fruitful, especially for applications to dark energy (and more general dark sector) models. One of the most intriguing developments involving imperfect fluids is the class of scalar-tensor theories that contain an essential mixing of scalar and tensor kinetic terms known as Kinetic Gravity Braiding [19–22], leading to a rich dark energy phenomenology. The imperfect fluid description in these works is based on the notion of chemical potential [21], which we are interested in here.

Naturally, since the main interest in the fluid description of minimally coupled scalar fields arises in cosmology, the theory of cosmological perturbations generated by scalar field matter is addressed in numerous works (e.g., [23–28] and references therein). Moreover, the effective field theory of relativistic media, including fluids, solids and exotic hypothetical media, has been developed in [29–35].

The fluid description of minimally coupled scalar fields crucially relies on the scalar field gradient being timelike, in order to be able to meaningfully define a fluid four-velocity. The analysis of scalars with non-timelike gradients has also been attempted [13, 18], but is still in its infancy.

In this work, we revisit the analogy between perfect fluid and minimally coupled scalar field which, albeit well-known, still leaves room for interesting developments. More specifically, a thermodynamical description of this fluid was recently presented [17], introducing the notions of temperature and chemical potential for the fluid. However, these results pose problems that we aim to solve. Addressing these issues also makes it possible to understand the analogy in a broader picture, by connecting it with the more general thermodynamical description of imperfect fluids in the context of scalar-tensor gravity [36–39]. This link additionally allows one to shed light on the Einstein frame formulation of first-order thermodynamics, which has so far remained elusive.

We first review some generalities to set the stage for our analysis. The action of gravity with a minimally coupled scalar field is

1 $$\begin{aligned} S=\int d^4 x \, \sqrt{-g} \left[ \frac{R}{2\kappa } + {{\mathscr {L}}}\left( \phi , X \right) \right] + S^\mathrm {(m)} \,, \end{aligned}$$

where^1^*g* is the determinant of the metric tensor $g_{ab}$, *R* is the Ricci scalar, $\kappa =8\pi G$, *G* is Newton’s constant,

2 $$\begin{aligned} X \equiv -\frac{1}{2} \, \nabla ^c \phi \nabla _c \phi \,, \end{aligned}$$

${{\mathscr {L}}}\left( \phi , X \right)$ is the scalar field Lagrangian density, and $S^\mathrm {(m)}$ describes matter other than the scalar field.

In the rest of this paper we assume that the scalar field has timelike gradient, $X>0$, and that this is future-oriented [41]. As is well-known (e.g., [12, 13, 42–46]), one can then establish an analogy between the scalar and a perfect fluid by taking the normalized gradient of $\phi$ as the fluid’s four-velocity,

3 $$\begin{aligned} u^a \equiv \frac{\nabla ^a \phi }{\sqrt{2X}} \,. \end{aligned}$$

Several works have derived the fluid-mechanical quantities corresponding to the minimally coupled scalar field [11–13, 16–22] and its thermodynamics has been studied recently in [17]. The effective $\phi$-fluid is a perfect fluid characterized by its four-velocity $u^a$, effective energy density $\rho$, pressure *P*, number density *n*, entropy density *s*, temperature *T*, and chemical potential $\mu$. The current state of knowledge of the scalar field-fluid correspondence is summarized by Eq. (3) and by the following dictionary appearing in Table 1 of [17]:

4 $$\begin{aligned} \rho= & {} 2X {{\mathscr {L}}}_X- {{\mathscr {L}}} \,, \end{aligned}$$

5 $$\begin{aligned} P= & {} {{\mathscr {L}}} \,, \end{aligned}$$

6 $$\begin{aligned} n= & {} \sqrt{2X}\, {{\mathscr {L}}}_X \,, \end{aligned}$$

7 $$\begin{aligned} \frac{s}{n}= & {} \phi \,, \end{aligned}$$

8 $$\begin{aligned} T= & {} \frac{ - {{\mathscr {L}}}_{\phi } }{ \sqrt{2X} \, {{\mathscr {L}}}_X} \,, \end{aligned}$$

9 $$\begin{aligned} \mu= & {} \frac{ 2X {{\mathscr {L}}}_X+\phi {{\mathscr {L}}}_{\phi } }{\sqrt{2X} \, \mathcal{L}_X } = \sqrt{2X} -\phi T \,, \end{aligned}$$

where ${{\mathscr {L}}}_{\phi } \equiv \partial {{\mathscr {L}}}/\partial \phi$ and ${{\mathscr {L}}}_X \equiv \partial {{\mathscr {L}}}/\partial X$. Assuming $\mathcal{L}_X>0$, *i.e.*, that the field $\phi$ is not a phantom, the particle number density *n* is non-negative. Further assuming the canonical Lagrangian density ${{\mathscr {L}}}=X-V$, also the energy density $\rho$ is non-negative.

Using Eqs. (3), (4), and (5), the stress-energy tensor of the scalar field

10 $$\begin{aligned} T_{ab}^{(\phi )} = {{\mathscr {L}}}_X \nabla _a \phi \nabla _b \phi +{{\mathscr {L}}} g_{ab} \,, \end{aligned}$$

which is conserved ($\nabla ^b T_{ab}^{(\phi )}=0$), is rewritten in the perfect fluid form

11 $$\begin{aligned} T_{ab}^{(\phi )} = \left( \rho +P\right) u_a u_b +P g_{ab} =\rho u_a u_b +P h_{ab} \,, \end{aligned}$$

where $h_{ab} \equiv g_{ab} +u_a u_b$ is the Riemannian metric on the 3-space orthogonal to $u^a$, satisfying

12 $$\begin{aligned} h_{ab} u^a =h_{ab} u^b=0 \end{aligned}$$

(${h^a}_b$ is the projection operator onto this 3-space).

The equation of motion for $\phi$

13 $$\begin{aligned} \nabla _a \Big ( {{\mathscr {L}}}_X \nabla ^a \phi \Big ) = -{{\mathscr {L}}}_{\phi } \end{aligned}$$

is written as

14 $$\begin{aligned} \nabla _a \Big ( nu^a \Big )\equiv \nabla _a N^a =-{{\mathscr {L}}}_{\phi } \,, \end{aligned}$$

which reduces to the familiar Klein-Gordon equation $\Box \phi =V_\phi$ if ${{\mathscr {L}}}\left( \phi , X \right) = X-V(\phi )$, where $V(\phi )$ is the scalar field potential.

If ${{\mathscr {L}}}={{\mathscr {L}}}(X)$, the scalar field theory is invariant under the shift symmetry $\phi \rightarrow \phi +C$ (where *C* is a constant) and there is a conserved Noether current

15 $$\begin{aligned} N^a = {{\mathscr {L}}}_X \nabla ^a \phi = n u^a \end{aligned}$$

satisfying

16 $$\begin{aligned} \nabla _a N^a=0 \,. \end{aligned}$$

$N^a$ is the analogue of the particle number current density. The particle number density in the comoving frame is the corresponding Noether charge

17 $$\begin{aligned} n = -N^0 = -u^c N_c = -\frac{\nabla ^a \phi }{\sqrt{2X}} \, \mathcal{L}_X \nabla _a \phi = \sqrt{2X} \, {{\mathscr {L}}}_X \,, \end{aligned}$$

consistently with Eq. (6). If ${{\mathscr {L}}}_{\phi } \ne 0$ (for example, if there is a potential $V(\phi )$), the analogue

18 $$\begin{aligned} N^a =n u^a \end{aligned}$$

of the particle current density is not conserved,^2^$\nabla _a N^a= -{{\mathscr {L}}}_{\phi } \ne 0$. Being derived from a scalar field, the $\phi$-fluid is, of course, irrotational (the kinematic quantities associated with the fluid four-velocity are computed in [44]).

Most of this analogy has been derived and validated in several situations and appears to be a special case of the more general equivalence between a scalar field coupled nonminimally to the Ricci scalar *R* and an effective *imperfect* fluid exhibiting heat conduction, bulk and shear viscosity, and anisotropic stresses [42–46]. The stress-energy tensor of the effective dissipative fluid in this more general case has the form

19 $$\begin{aligned} T_{ab}^\mathrm {(dissipative)} = \rho u_a u_b +P h_{ab} +q_a u_b + q_b u_a +\pi _{ab} \,, \end{aligned}$$

where

20 $$\begin{aligned} P= P_\mathrm {non-viscous} +P_\textrm{viscous} \end{aligned}$$

is the sum of a non-viscous and of a viscous pressure (the latter is associated with bulk viscosity), $q^a$ is a purely spatial (*i.e.*, $q_c u^c=0$) heat flux density, and $\pi _{ab}$ (with ${\pi ^a}_a=0$, $\pi _{ab}u^a =\pi _{ab} u^b=0$) denotes the anisotropic stresses. If the scalar field $\phi$ is minimally coupled to *R*, all the imperfect fluid terms vanish and Eq. (19) reduces to the perfect fluid form (11). This analogy has been often studied in the context of FLRW cosmology and of the special theory of conformally/nonminimally coupled scalar fields, but has seldom been considered for general “first-generation” scalar-tensor gravity [43, 44]. More recently, it has been extended to Horndeski gravity [45, 46].

The introduction of temperature *T* and chemical potential $\mu$ in the correspondence between minimally coupled scalar field and perfect fluid is quite recent (appearing only in [17] to the best of our knowledge) and has not been tested as well as the rest of the analogy. Indeed, the derivation of *T* and, as a consequence, of $\mu$ in [17] exhibits an inconsistency (that does not affect the other fluid quantities), that we correct here. Before analysing the details (Sect. 2), it is already apparent that *T* and $\mu$ given by Eqs. (8) and (9) suffer from three problems.

1. The first issue (already noted in [17]) is that both *T* and $\mu$ can be negative. This fact is surprising because, contrary to the nonminimally coupled scalars of scalar-tensor gravity, the effective $\phi$-fluid is otherwise well-behaved and satisfies the weak and null energy conditions, hence one expects *T* and $\mu$ to be non-negative like $\rho$ and *n*.
2. The most serious problem is that, according to Eq. (8), there is a temperature gradient. Moreover, in general the effective $\phi$-fluid is non-geodesic, with non-zero acceleration
  21 $$\begin{aligned} {\dot{u}}_a\equiv & {} u^c \nabla _c u_a = - \frac{1}{{2 X}} \left( \nabla _a X + \frac{\nabla _c X \nabla ^c \phi }{2 X} \, \nabla _a \phi \right) \,. \end{aligned}$$
  Then, there must necessarily be a heat current with density [48]
  22 $$\begin{aligned} q_a = - K \left( h_{ab}\nabla ^b T + T {\dot{u}}_a \right) \,, \end{aligned}$$
  where *K* is the (analogue of) the thermal conductivity. This generalized Fourier law is one of the three constitutive equations of Eckart’s first-order thermodynamics [48] and a minimal assumption. The first term in the right-hand side of Eq. (22) is nothing but the usual Fourier law, while the second one is a purely relativistic “inertial” contribution discovered by Eckart [48]. The heat conduction described by $q_a$ makes a fluid dissipative and endows its stress-energy tensor with the dissipative terms appearing in Eq. (19). Then, how can the fluid equivalent of a minimally coupled $\phi$ be a perfect fluid described by (11)? A heat current would necessarily show up in the comoving (or Eckart) frame based on the four-velocity (3). The *only* way for $q_a$ to vanish identically is if $T = 0$.
3. Although here we limit ourselves to scalar fields coupled minimally to *R*, from the perspective of the broader nonminimally coupled scalar field thermodynamics (which is still under development but has certain firm points) the fact that a minimally coupled scalar field fluid is endowed with a non-zero temperature appears very odd. In that context [36–39, 46], the nonminimal coupling with *R* is responsible for a nonvanishing fluid temperature, therefore the fluid equivalent to a minimally coupled $\phi$ and with Lagrangian depending only on $\phi$ and *X* should always have zero temperature.

In the rest of this article we address these problems. We begin by correcting the temperature (8), establishing the fact that the fluid equivalent to a minimally coupled $\phi$ has always zero temperature, resolving the first conundrum of scalar-tensor thermodynamics. As a consequence, only the first term in the chemical potential $\mu = \sqrt{2X}-\phi T$ remains, which makes this quantity positive-definite. The second and third issue listed above are also solved because the heat flux density $q_a$ then vanishes identically and the fluid becomes non-dissipative.

After describing the thermodynamics of the fluid equivalent to a minimally coupled $\phi$, we are also able to comment on the thermodynamics of phantom scalar fields with ${{\mathscr {L}}}_X<0$, which have been the subject of an extensive literature, in conjunction with studies considering the possibility of a very negative equation of state ($w \equiv P/\rho <-1$) for the dark energy driving the present acceleration of the cosmic expansion, e.g. [50–52]. Although the claims of a phantom equation of state are disputed, the possibility of $w<-1$ is not excluded by present cosmological observations [53]. Phantoms are unstable from the classical, and even more from the quantum, point of view but they are still accepted in the cosmological literature as the expression of a truncated action that will be cured if all terms are included. The literature on phantom field thermodynamics, now mostly a decade old, has not been conclusive and we contribute to a clearer picture.

Finally, we can extend the $\phi$-fluid correspondence to include scalar fields coupled nonminimally *to matter* (but not to *R*). The effect of these couplings is analogous to that of a scalar field potential which constitutes a source of fluid “particles” making the “particle number density” *n* a non-conserved quantity, but has no drastic effect on the rest of the analogy. This extension allows one to discuss the Einstein frame version of scalar-tensor gravity in which the gravitational Brans-Dicke-like field ${\tilde{\phi }}$ couples explicitly to matter but not to *R* (contrary to the Jordan frame formulation of the same theory in which the scalar $\phi \ne {\tilde{\phi }}$ couples to *R* but not to matter). This development makes it possible to fill a gap in the first-order thermodynamics of scalar-tensor gravity which, being based on the notion of temperature, was thus far unable to deal with the Einstein frame description.

## Temperature and chemical potential in the scalar field-fluid analogy

Consider the first law of thermodynamics [54, Box 22.1, p. 561])

23 $$\begin{aligned} d \left( \frac{\rho }{n} \right) + P \, d \left( \frac{1}{n} \right) = T \, d \left( \frac{s}{n} \right) \, , \end{aligned}$$

where *T* denotes the temperature, *s* the entropy per unit volume (*i.e.*, $s = S/V$), $\rho$ is the internal energy density (per unit volume), and *P* is the pressure. The symbol *s* in [54] corresponds to *s*/*n* in our discussion.

Now, taking *s* and *n* as independent variables from [54, Box 22.1, p. 561], one has that

24 $$\begin{aligned} T (s, n) = \frac{1}{n} \frac{\partial \rho }{\partial (s/n)} \Big |_n = \frac{\partial \rho }{\partial s} \Big |_n \quad , \end{aligned}$$

thus, since Eq. (10) maps into a perfect fluid, the absence of any dissipative effects, hence vanishing heat fluxes, requires $$ T = 0$$, in accordance with the principles of the thermodynamics of scalar-tensor gravity [36–39, 46]. Therefore, assuming $$\phi = \phi (s,n)$$ and $$X = X (s,n)$$, one has that

25 $$\begin{aligned} 0 = \frac{\partial \rho }{\partial s} \Big |_n = - \mathscr {L}_{\phi } \,\frac{\partial \phi }{\partial s} \Big |_n \quad , \end{aligned}$$

where we have taken advantage of Eqs. (4) and (6). The condition in Eq. (25) is then satisfied if $\mathscr {L}_{\phi } = 0$ or $\frac{\partial \phi }{ \partial s} \Big |_n = 0$. Since, in general, $\mathscr {L}$ will contain a potential term, consistency with the thermodynamics of scalar-tensor gravity [36–39, 46] translates into the condition

26 $$\begin{aligned} \frac{\partial \phi }{\partial s} \Big |_n = 0 \, , \end{aligned}$$

so that the temperature of gravity vanishes for a scalar field non-minimally coupled to Einstein gravity.

On a similar note, it is easy to see that combining [54, Box 22.1, p. 561]

27 $$\begin{aligned} P \left( n, s \right) = n\, \frac{\partial \rho }{\partial n} \Big |_{s/n} -\rho \, , \end{aligned}$$

with Eqs. (4) and (5) recovers the perfect fluid identification $P = \mathscr {L}$ if and only if

28 $$\begin{aligned} \frac{\partial \phi }{\partial n}\Big |_{s/n} = 0 \, , \end{aligned}$$

when $$\mathscr {L}_\phi \ne 0$$.

It is then easy to identify the chemical potential of the system, which reads [54, Box 22.1, p. 561])

29 $$\begin{aligned} \mu = \frac{P + \rho }{n} = \sqrt{2X} \, , \end{aligned}$$

where we have again taken advantage of Eqs. (4)–(6).

The condition in Eq. (7) is incompatible with both the thermodynamic analogy presented here and the requirement of conservation of the entropy per particle along perfect fluid lines (see Appendix 1). This condition is, however, marginal in our discussion since it is not used.

### Approach to the diffusive equilibrium

One can a posteriori derive an equation describing the approach to diffusive equilibrium along the fluid lines. For relativistic fluids, the chemical potential $\mu$ and the (purely spatial) diffusive flux density of particles $q_a^{(p)}$ will obey a generalization of Fick’s law analogous to Eckart’s generalization (22) of Fourier’s law (cf. Ref. [55])

30 $$\begin{aligned} q_a^{(p)} = -{{\mathscr {D}}} \left( h_{ab}\nabla ^b \mu + \mu \, {\dot{u}}_a \right) \,, \end{aligned}$$

where ${{\mathscr {D}}}$ is a diffusion coefficient analogous to the thermal conductivity *K*. Diffusive equilibrium is reached when the chemical potential $\mu$ vanishes identically (in the presence of acceleration ${\dot{u}}^a$, a constant $\mu$ would still generate particle diffusion due to the second term in the right-hand side of Eq. (30). Equation (30) is reminiscent of a relativistic version of the drift-diffusion equation [56]. This is used, for example, in the context of semiconductors [57], where it describes particle currents (for electrons and holes) in terms of the particle density gradients and a term containing the electric field vector.^3^

Let us compute the derivative $d\mu / d\tau$, where $\tau$ is the proper time along the flow lines of the effective $\phi$-fluid:

31 $$\begin{aligned} \frac{d\mu }{d\tau } \equiv u^c \nabla _c \mu = \frac{\nabla ^c \phi }{\sqrt{2X}} \, \nabla _c \left( \sqrt{2X} \right) = \frac{\nabla ^c \phi \, \nabla _c X}{2X} \,. \end{aligned}$$

Now use the expression of the expansion scalar of the $\phi$-fluid [44, 46]

32 $$\begin{aligned} \varTheta \equiv \nabla _a u^a = \frac{1}{\sqrt{2 X}} \left( \Box \phi - \frac{\nabla _c X \nabla ^c \phi }{2 X} \right) \end{aligned}$$

to eliminate the term containing second derivatives of $\phi$ in Eq. (31), obtaining

33 $$\begin{aligned} \frac{d\mu }{d\tau } = -\mu \, \varTheta +\Box \phi \,. \end{aligned}$$

This equation is not so simple because of the d’Alembertian of $\phi$ in the right-hand side. However, to gain some insight, we can consider the situation in which ${{\mathscr {L}}}$ does not depend on $\phi$, for example a free scalar field with ${{\mathscr {L}}}=X$, in which case $\Box \phi =0$ and Eq. (33) reduces to^4^

34 $$\begin{aligned} {\dot{\mu }} = -\mu \, \varTheta \,. \end{aligned}$$

One can introduce a representative length $\ell$ by [58]

35 $$\begin{aligned} \frac{ {\dot{\ell }}}{\ell } \equiv \frac{ \varTheta }{3} \end{aligned}$$

and then

36 $$\begin{aligned} \frac{ {\dot{\mu }}}{\mu } = -\frac{3{\dot{\ell }}}{\ell } \end{aligned}$$

so that $\mu = \text{ const. }/\ell ^3$. The simplified evolution equation of $\mu$ then simply says that when the flow expands and dilutes, $\mu$ decreases and the state of equilibrium $\mu =0$ is approached, while when the flow gets concentrated, the chemical potential increases and there is departure from the equilibrium state. In particular, the $\phi$-flow is diluted in an expanding universe, which will approach the diffusive equilibrium state $\mu =0$ as $\ell \rightarrow +\infty$. Near spacetime singularities, instead, this flow is focused, the flow lines become closer and closer, and there are extreme departures from the equilibrium state $\mu =0$. In principle, this understanding of the approach to equilibrium in the thermodynamical picture based on $\mu$ is equivalent to that obtained in the context of scalar-tensor thermodynamics based on *T* [36–39]. However, in the comoving frame one does not see particle diffusion, as explained in the next subsection. When the second term $\Box \phi$ is included in the right-hand side of Eq. (33), the situation becomes more complex since this term could in principle be positive or negative, hence it can favour the approach to equilibrium or oppose it depending on its sign.

### No diffusive particle current in the comoving frame

The effective stress-energy tensor (10) of the effective $\phi$-fluid has the perfect fluid form (11), yet the chemical potential $\mu = \sqrt{2X}$ depends on the spacetime position so its variation must give rise to a diffusive $\phi$- (or “particle”) current (the acceleration also contributes to this diffusive flow according to Eq. (30)). Then it is natural to ask why we do not see a vector $q^a_\mathrm {(p)}$ describing this diffusion in the effective fluid stress-energy tensor. The answer is well-known to researchers working with relativistic dissipative fluids, in which the direction of the energy flow is distinct from that of the particle flow. In dissipative fluids, the Eckart (or comoving) frame is based on the particle four-velocity (that is, the four-velocity of the Eckart or comoving observers is the $u^a$ of the fluid given by Eq. (3)). Since this frame is adapted to follow the total flow of particles, the diffusive particle current vanishes. The Landau or energy frame, instead, is the frame of observers with four-velocity $u^a_\mathrm {(L)} \ne u^a$ moving with the energy flow. In this frame, Landau observers see a diffusive particle flow described by a current $q^a_\mathrm {(p)}$ but not an energy flow, since the heat current density $q^a_\mathrm {(L)}$ vanishes identically. For a perfect fluid, the Eckart (comoving) and the Landau (energy) frames coincide and both the heat and the particle diffusion currents are zero.

We have shown that $T = 0$ but $\mu =\sqrt{2X}\ne 0$ in the comoving frame of the effective fluid associated with a minimally coupled scalar field. Here we check explicitly that this fact does not contradict the vanishing of the diffusion current because the two terms in the right hand side of Eq. (30) cancel each other out. We have

37 $$\begin{aligned} \nabla _a\mu = \nabla _a \left( \sqrt{2X} \right) = \frac{\nabla _a X}{\sqrt{2X}} \end{aligned}$$

and the spatial gradient of $\mu$ is

38 $$\begin{aligned} h_{ab}\nabla ^b \mu= & {} \left( g_{ab} + \frac{\nabla _a \phi \nabla _b \phi }{2 X} \right) \, \frac{\nabla ^b X}{\sqrt{2X}} \nonumber \\{} & {} \nonumber \\= & {} \frac{\nabla _a X}{\sqrt{2X}} + \frac{\nabla _b \phi \nabla ^b X}{(2 X)^{3/2}} \, \nabla _a \phi \,. \end{aligned}$$

Adding to this quantity the acceleration term $\mu \, {\dot{u}}_a$ and using the expression (21) of the acceleration yields

39 $$\begin{aligned} h_{ac} \nabla ^c \mu + \mu \, {\dot{u}}_a = 0 \,, \end{aligned}$$

which ensures that there is no diffusive “particle” current in this frame in spite of the non-uniform chemical potential.

### Phantom fields

A phantom scalar field is obtained from a canonical one by changing the sign with which *X* appears in the Lagrangian,

40 $$\begin{aligned} {{\mathscr {L}}}=-X-V(\phi ) \,, \end{aligned}$$

which changes the usual stress-energy tensor to

41 $$\begin{aligned} T_{ab} = -\nabla _a \phi \nabla _b \phi + \frac{1}{2} \, g_{ab} \nabla ^c\phi \nabla _c \phi - g_{ab} V \end{aligned}$$

and the equation of motion to

42 $$\begin{aligned} \Box \phi +V_\phi =0 \,. \end{aligned}$$

For example, in FLRW cosmology (which is no doubt the main area of theoretical physics contemplating phantom fields) $\phi =\phi (t)$ and $X>0$, together with $V(\phi ) \ge 0$. The phantom equation of state parameter is then

43 $$\begin{aligned} w \equiv \frac{P}{\rho } = \frac{ {{\mathscr {L}}}}{ -2X-{{\mathscr {L}}}} = \frac{-X-V}{-X+V} = -1 -\frac{2X}{V-X} \end{aligned}$$

and the requirement $$\rho >0 $$, equivalent to $$ 0< X< V$$, then yields $$w<-1$$.

According to the previous section, for a phantom scalar field the particle number density, temperature, and chemical potential are

44 $$\begin{aligned} n= & {} - \sqrt{2X} <0 \,,\end{aligned}$$

45 $$\begin{aligned} T= & {} 0 \,,\end{aligned}$$

46 $$\begin{aligned} \mu= & {} \sqrt{2X} >0 \,. \end{aligned}$$

While *T* and $\mu$ remain the same as for non-phantom fields, the number density *n* becomes negative for phantoms.

The thermodynamics of phantom fields and, more in general, phantom fluids, has been discussed in many works, usually beginning with assumptions different from ours and often assuming negative temperature (or entropy) and positive chemical potential (or entropy), or *vice-versa*, from the outset and often considering quantum fields [59–71]. Usually, these discussions are limited to FLRW cosmology, where negative temperatures were speculated independently [72]. It is difficult to compare all these different (and sometimes contradictory) scenarios and assumptions, and to make sense of their conclusions caused by such an exotic, and most likely unphysical, field as the phantom. Moreover, our analogy is restricted to classical scalar fields. However, the picture that we offer for nonminimally coupled scalars in GR seems more grounded in fluid physics than many scenarios in the literature, in the sense that temperature and chemical potential are well-defined, with $$ T=0$$ and $$\mu >0$$, but $$n <0$$. This feature is definitely unphysical, as are many of the consequences of a phantom field permeating the universe, and we will not consider phantom fields further.

## Nonminimal coupling to matter and Einstein frame formulation of scalar-tensor gravity

Let us consider now the situation in which the scalar field couples nonminimally to other forms of matter, which are described by the Lagrangian density $\mathcal{L}^\mathrm {(m)}$ through a coupling function $f(\phi )$ (this coupling is non-trivial if $f(\phi )\ne$ const.). For simplicity, we restrict to the scalar field Lagrangian ${{\mathscr {L}}}=X-V(\phi )$. The total Lagrangian density is then

47 $$\begin{aligned} {{\mathscr {L}}}= X- V(\phi ) +f(\phi ) {{\mathscr {L}}}^\mathrm {(m)} \,. \end{aligned}$$

The equation of motion of $\phi$ becomes

48 $$\begin{aligned} \Box \phi =V_\phi - f_\phi {{\mathscr {L}}}^\mathrm {(m)} \,. \end{aligned}$$

The extra term acts as a source of $\phi$, hence as a source of “particles” in the effective $\phi$-fluid. As a consequence, the stress-energy tensors of $\phi$ and of the other matter are not conserved ($\nabla ^b T_{ab}^{(\phi )} \ne 0$, $\nabla ^b T_{ab}^\mathrm {(m)} \ne 0$) but their sum is, $\nabla ^b \left( T_{ab}^{(\phi )} + T_{ab}^\mathrm {(m)}\right) = 0$. The coupling term on the right-hand side of Eq. (48) acts in the same way as the potential $V(\phi )$, preventing the conservation of the “particle” current density $N^a = nu^a=\nabla ^a\phi$ according to Eq. (16). Indeed, this extra term breaks the shift invariance $\phi \rightarrow \phi +C$ of the scalar field Lagrangian $\mathcal{L}={{\mathscr {L}}}(X)$ in the absence of a potential $V(\phi )$ and prevents $N^a$ from being a conserved Noether current even when $V(\phi ) \equiv 0$.

Since in the Einstein frame the scalar couples minimally to gravity but nonminimally to matter, these considerations open up the possibility of discussing the Einstein frame formulation of the thermodynamics of scalar-tensor gravity, which has so far been developed in the Jordan frame [36–39].

First-order thermodynamics deals with theories that have (Jordan frame) action

49 $$\begin{aligned} S_\textrm{ST}= & {} \frac{1}{16\pi } \int d^4x \sqrt{-g} \left[ \phi R -\frac{\omega (\phi )}{\phi } \, \nabla ^c\phi \nabla _c\phi -V(\phi ) \right] \nonumber \\{} & {} \nonumber \\&\,&+ S^\mathrm{(m)}, \end{aligned}$$

where the Brans-Dicke scalar $\phi >0$ is approximately the inverse of the effective gravitational coupling $G_\textrm{eff}$ and $\omega (\phi )$ is the “Brans-Dicke coupling”. The scalar contribution to the energy-momentum tensor can be cast in the form of an effective imperfect fluid (19) [44]. Applying Eckart’s first-order non-equilibrium thermodynamics to this fluid allows one to recover an effective temperature *T* (and thermal conductivity *K*)

50 $$\begin{aligned} K T = \frac{ \sqrt{-\nabla ^c \phi \nabla _c \phi }}{ 8 \pi \phi } \,. \end{aligned}$$

This is nothing but a temperature relative to GR, which represents the $$ KT=0$$ equilibrium state. In this way, one can depict a landscape of gravity theories, where different theories (or classes thereof) are identified by their temperature relative to equilibrium and obtain an understanding of how this equilibrium might be approached through a dissipation process. However, the Einstein frame could not be handled in this picture based on the notion of temperature. The alternative and complementary picture relying on the chemical potential that was developed in the previous sections, on the other hand, can fill the gap. We switch from the Jordan to the Einstein frame by performing the well-known conformal transformation of the metric [73]

51 $$\begin{aligned} g_{ab} \rightarrow {\tilde{g}}_{ab} \equiv \phi \, g_{ab} \end{aligned}$$

and the scalar field redefinition $\phi \rightarrow {\tilde{\phi }}$ with

52 $$\begin{aligned} d{\tilde{\phi }} = \sqrt{ \frac{| 2\omega +3|}{16\pi }} \, \frac{d\phi }{\phi } \,. \end{aligned}$$

The action then reads

53 $$\begin{aligned} S_\text {EF}= & {} \int d^4x \sqrt{-g} \left[ \frac{ {\tilde{R}}}{16\pi } -\frac{1}{2} \, {\tilde{g}}^{ab} \, \nabla _a{\tilde{\phi }} \nabla _b{\tilde{\phi }} -U({\tilde{\phi }}) \right. \nonumber \\{} & {} \nonumber \\&\,&\left. +\frac{{{\mathscr {L}}}^\text {(m)}}{\phi ^2({\tilde{\phi }})}\right] \,, \end{aligned}$$

with

54 $$\begin{aligned} U ({\tilde{\phi }}) = \frac{V(\phi )}{16\pi \phi ^2}\left| _{\phi =\phi ( {\tilde{\phi }}) } \right. \,. \end{aligned}$$

All Einstein frame variables $\left( {\tilde{g}}_{ab}, {\tilde{\phi }} \right)$ are denoted by a tilde. The Einstein frame field equations read

55 $$\begin{aligned}{} & {} {\tilde{R}}_{ab}-\frac{1}{2} \, {\tilde{g}}_{ab} {\tilde{R}} = 8\pi \left( \text{ e}^{- \sqrt{\frac{64\pi }{|2\omega +3|}} \, {\tilde{\phi }} } \, T_{ab}^\mathrm{(m)} + \nabla _a {\tilde{\phi }} \nabla _b {\tilde{\phi }} \right. \nonumber \\{} & {} \quad \left. -\frac{1}{2} \, {\tilde{g}}_{ab}\, {\tilde{g}}^{cd} \nabla _c {\tilde{\phi }} \nabla _d {\tilde{\phi }} - U({\tilde{\phi }}) \, {\tilde{g}}_{ab} \right) ,\nonumber \\ \end{aligned}$$

56 $$\begin{aligned}{} & {} {\tilde{g}}^{ab} \nabla _a \nabla _b {\tilde{\phi }} -\frac{dU}{d{\tilde{\phi }}}\nonumber \\{} & {} \quad +8\, \sqrt{ \frac{\pi }{|2\omega +3|}} \, \text{ e}^{ -\sqrt{\frac{64\pi }{|2\omega +3|}}\, {\tilde{\phi }} } \, \mathcal{L}^\mathrm{(m)} = 0 \,. \end{aligned}$$

The scalar contribution to the stress-energy tensor arising from this action is of course that of a perfect fluid (11). However, this presents a puzzle for the first-order thermodynamics of scalar-tensor theories. The thermodynamical formalism based on the temperature description is not suitable for a perfect fluid, since all imperfect fluid quantities vanish and the theory becomes trivial. This means that the approach to equilibrium cannot be studied. The formalism based on temperature only works for gravitational theories in representations where an effective imperfect fluid description can be found, which is possible only if the scalar is directly coupled to *R* in the action. These considerations relate to the well-known but hard-to-tackle problem of the ambiguity that arises in distinguishing between “gravitational” and “matter” degrees of freedom whenever we switch representation through a conformal transformation [74].

However, the notion of chemical potential comes to the rescue. Although the temperature *T* of the Einstein frame scalar field effective fluid is zero, according to the previous sections, the chemical potential ${\tilde{\mu }} = \sqrt{ 2{\tilde{X}}}$ is not. Now the scalar field ${\tilde{\phi }}$ has gravitational nature and is always present in spacetime, that is, one cannot decide to set it to zero or replace it with other forms of matter. The state of diffusive equilibrium corresponds to ${\tilde{\mu }}=0$ and ${\tilde{\phi }}=$ const., but this condition automatically recovers GR (possibly, with a cosmological constant if a potential for the scalar field is present), as a limiting case given that a timelike gradient for the scalar field represents our starting assumption for this analogy. This result goes hand-in-hand with that of first-order thermodynamics of scalar-tensor gravity formulated in the Jordan frame, where GR is the zero-temperature state of equilibrium [36–38, 46]. In the Einstein frame, instead, ${\tilde{K}} {\tilde{T}}$ is identically zero but GR is the state of equilibrium of scalar-tensor gravity corresponding to vanishing chemical potential ${\tilde{\mu }}=\sqrt{2{\tilde{X}}}$. Therefore, the thermodynamical picture based on the chemical potential solves the issue and an understanding of the approach to equilibrium even for theories described by perfect fluids is possible. We would argue that the simplicity of the argument adds to the first-order thermodynamics of scalar-tensor gravity instead of detracting from it.

## Conclusions

We have begun our discussion with minimally coupled scalar fields in GR and have corrected the current view of the analogy between these fields and effective perfect fluids with regard to temperature and chemical potential. This new view has allowed us to reformulate the first-order thermodynamics of scalar-tensor gravity as seen from the Einstein conformal frame, which was not possible earlier. Hence, we conclude this work from the broader view of the equivalent fluid of a scalar field nonminimally coupled to *R* in scalar-tensor gravity. Two main conclusions emerge.

First, if the scalar field $\phi$ described by a Lagrangian density ${{\mathscr {L}}}\left( \phi , X \right)$ couples nonminimally with the Ricci scalar in the Jordan frame description of scalar-tensor gravity, the equivalent fluid has a nonvanishing temperature defined in [36, 37, 44, 46] (exceptions are theories in which the scalar field is non-dynamical [47]).

Second, in GR a scalar field coupled minimally to *R* (but possibly nonminimally to other forms of matter) has zero temperature *T* but nonvanishing chemical potential $\mu =\sqrt{2X}$. However, in the comoving (or Eckart) frame, no diffusive flux of “$\phi$-particles” is visible because this frame follows the total motion of this effective fluid. This situation includes the Einstein frame description of scalar-tensor gravity and allows one to establish that GR is the state of diffusive equilibrium (*i.e.*, $\mu \equiv 0$) of scalar-tensor gravity formulated in the Einstein frame. The previous approaches to first-order thermodynamics of scalar-tensor and Horndeski gravity [36–39, 46] were based on the Jordan frame description and on the *temperature* of the $\phi$-fluid and were thus unable to deal with the Einstein frame. Realizing that scalar fields minimally coupled to the curvature should have zero temperature but non-zero *chemical potential* is the key to resolve that conundrum.

This work is limited to situations in which there are no derivative couplings of the scalar and no second derivatives of $\phi$ in the Lagrangian, while theories with ${{\mathscr {L}}}={{\mathscr {L}}}\left( \phi , X, \Box \phi \right)$ are the subject of much attention in the literature. Other questions arise naturally: what is the Landau frame for the fluid equivalent of a nonminimally coupled scalar field? The discussion in the literature thus far has exclusively been based on the comoving (Eckart) frame, but the choice of the Landau frame is advantageous in the analysis of relativistic fluids in nuclear collisions [75–79] and may disclose unexpected view of scalar fields as well. Future work will focus on these questions.

## Data Availability

This manuscript has no associated data or the data will not be deposited. [Authors’ comment: There are no data associated with this work because of its theoretical and mathematical nature.]

## Appendix A: $s/n=$ const. along perfect fluid lines

Here we reproduce a standard result of perfect fluids stating that the entropy per particle *s*/*n* is constant along the flow lines of a perfect fluid in which entropy and particle number are conserved. As a consequence, neighbouring fluid lines do not exchange entropy per particle and different values of *s*/*n* distinguish different flow lines.

Consider a flow line with four-tangent $u^a$ parametrized by the proper time $\tau$; we have

A.1 $$\begin{aligned} \frac{d}{d\tau } \left( \frac{s}{n} \right) = \frac{1}{n} \, \frac{ds}{d\tau } - \frac{s}{n^2}\, \frac{dn}{d\tau } \,. \end{aligned}$$

Conservation of particle number gives

A.2 $$\begin{aligned} 0=\nabla _c N^c = \nabla _c \left( nu^c \right) =n\nabla _c u^c + u^c \nabla _c \, n \end{aligned}$$

and

A.3 $$\begin{aligned} \frac{dn}{d\tau } \equiv u^c\nabla _c n =- n \nabla _c u^c \,. \end{aligned}$$

Likewise, conservation of entropy for a perfect fluid without dissipation yields

A.4 $$\begin{aligned} 0=\nabla _c s^c = \nabla _c \left( su^c \right) =s\nabla _c u^c + u^c \nabla _c \, s \end{aligned}$$

and

A.5 $$\begin{aligned} \frac{dn}{d\tau } =- s \nabla _c u^c \,. \end{aligned}$$

Then we have

A.6 $$\begin{aligned} \frac{d}{d\tau } \left( \frac{s}{n} \right)= & {} \frac{1}{n} \left( \frac{ds}{d\tau } -\frac{s}{n} \, \frac{dn}{d\tau } \right) \nonumber \\{} & {} \nonumber \\= & {} \frac{1}{n} \left( -s\nabla _c u^c + \frac{s}{n} \, n \nabla _a u^a \right) =0 \,. \end{aligned}$$

For the effective $\phi$-fluid, *s*/*n* cannot be identified with the scalar field $\phi$, as done in previous literature. Indeed, as shown above, *s*/*n* is constant along fluid lines (this is certainly the case also for the effective $\phi$-fluid if ${{\mathscr {L}}}_{\phi }=0$), while $\phi$ necessarily changes along the flow lines. In fact, in general, $\phi$ depends on the proper time $\tau$ along the flow lines, as well as on the spatial coordinates of the 3-space orthogonal to these flow lines. Indeed, in applications to FLRW cosmology (the main purpose of Refs. [19–22]), $\phi$ depends *only* on $\tau$, which coincides with the FLRW comoving time. The situation in which $\phi$ depends only on *s*/*n* and, in particular, the identification $\phi =s/n$ would mean that there is a coordinate system in which the gradient $\nabla _a \phi$ is purely spatial, then the latter is spacelike and cannot be timelike, which is instead essential for identifying (3) with the effective fluid four-velocity and constructing the effective fluid description of the scalar field.

## Footnotes

1 We follow the notation of Ref. [40]. The metric signature is $({-}{+}{+}{+})$ and units are used in which Newton’s constant *G* and the speed of light *c* are unity.

2 In a dissipative fluid, the directions of the particle flow and of the energy flow are different. As a consequence, $N^a$ coincides with $n u^a$ in the comoving (or Eckart) frame [48] which is adapted to follow the total flux of particles, while $N^a=nu^a + v_{(L)}^a$ in the Landau (or energy frame) [49], where $v_{(L)}^a$ is the diffusive current density of particles caused by gradients of the chemical potential $\mu$. The Eckart and the Landau frames coincide for a perfect fluid, which is the case we are interested in.

3 The similar form of the drift current and of Eckart’s heat current density is due to the presence of a force, hence of an acceleration, in the constitutive law leading to the expression of the drift current. This similarity could potentially be of interest for analogue gravity.

4 If ${{\mathscr {L}}}_X=1$, then $n=\mu$ satisfies the same equation, which is reported in [35].

## References

[1] Amendola L, Tsujikawa S (2010). Dark Energy, Theory and Observations.

[2] Peebles PJE, Ratra B (2003). The Cosmological Constant and Dark Energy. Rev. Mod. Phys..

[3] Armendariz-Picon C, Damour T, Mukhanov VF (1999). k - inflation. Phys. Lett. B.

[4] Armendariz-Picon C, Mukhanov VF, Steinhardt PJ (2001). Essentials of k essence. Phys. Rev. D.

[5] Bilic N (2008). Thermodynamics of k-essence. Phys. Rev. D.

[6] Padmanabhan T, Choudhury TR (2002). Can the clustered dark matter and the smooth dark energy arise from the same scalar field?. Phys. Rev. D.

[7] Sen A (2002). Field theory of tachyon matter. Mod. Phys. Lett. A.

[8] Sen A (2002). Rolling tachyon. JHEP.

[9] Sen A (2002). Tachyon matter. JHEP.

[10] Gibbons GW (2003). Thoughts on tachyon cosmology. Class. Quant. Grav..

[11] Unnikrishnan S, Sriramkumar L (2010). A note on perfect scalar fields. Phys. Rev. D.

[12] Faraoni V (2012). The correspondence between a scalar field and an effective perfect fluid. Phys. Rev. D.

[13] Semiz I (2012). Comment on ‘Correspondence between a scalar field and an effective perfect fluid. Phys. Rev. D.

[14] Bamba K, Capozziello S, Nojiri S, Odintsov SD (2012). Dark energy cosmology: the equivalent description via different theoretical models and cosmography tests. Astrophys. Space Sci..

[15] Diez-Tejedor A, Feinstein A (2005). Relativistic hydrodynamics with sources for cosmological K-fluids. Int. J. Mod. Phys. D.

[16] Diez-Tejedor A (2013). Note on scalars, perfect fluids, constrained field theories, and all that. Phys. Lett. B.

[17] Piattella OF, Fabris JC, Bilić N (2014). Note on the thermodynamics and the speed of sound of a scalar field. Class. Quant. Grav..

[18] C. Gergely, Z. Keresztes, L. Á. Gergely, Minimally coupled scalar fields as imperfect fluids. Phys. Rev. D **102**(2), 024044 (2020). 10.1103/PhysRevD.102.024044. arXiv:2007.01326 [gr-qc]

[19] Deffayet C, Pujolas O, Sawicki I, Vikman A (2010). Imperfect Dark Energy from Kinetic Gravity Braiding. JCAP.

[20] Lim EA, Sawicki I, Vikman A (2010). Dust of Dark Energy. JCAP.

[21] Pujolas O, Sawicki I, Vikman A (2011). The Imperfect Fluid behind Kinetic Gravity Braiding. JHEP.

[22] Mirzagholi L, Vikman A (2015). Imperfect Dark Matter. JCAP.

[23] Khlopov M, Malomed BA, Zeldovich IB (1985). Gravitational instability of scalar fields and formation of primordial black holes. Mon. Not. Roy. Astron. Soc..

[24] Garriga J, Mukhanov VF (1999). Perturbations in k-inflation. Phys. Lett. B.

[25] Fabris JC, Goncalves SVB, Tomimura NA (2000). An Analysis of cosmological perturbations in hydrodynamical and field representations. Class. Quant. Grav..

[26] Arroja F, Sasaki M (2010). A note on the equivalence of a barotropic perfect fluid with a K-essence scalar field. Phys. Rev. D.

[27] Sawicki I, Saltas ID, Amendola L, Kunz M (2013). Consistent perturbations in an imperfect fluid. JCAP.

[28] Christopherson AJ, Hidalgo JC, Malik KA (2013). Modelling non-dust fluids in cosmology. JCAP.

[29] Dubovsky S, Gregoire T, Nicolis A, Rattazzi R (2006). Null energy condition and superluminal propagation. JHEP.

[30] Dubovsky S, Hui L, Nicolis A, Son DT (2012). Effective field theory for hydrodynamics: thermodynamics, and the derivative expansion. Phys. Rev. D.

[31] Nicolis A, Penco R, Piazza F, Rattazzi R (2015). Zoology of condensed matter: Framids, ordinary stuff, extra-ordinary stuff. JHEP.

[32] G. Ballesteros, D. Comelli, L. Pilo, Massive and modified gravity as self-gravitating media. Phys. Rev. D **94**(12), 124023 (2016). 10.1103/PhysRevD.94.124023. arXiv:1603.02956 [hep-th]

[33] A. Nicolis, R. Penco, R. A. Rosen, Relativistic fluids, superfluids, solids and supersolids from a coset construction. Phys. Rev. D **89**(4), 045002 (2014). 10.1103/PhysRevD.89.045002. arXiv:1307.0517 [hep-th]

[34] Ballesteros G (2015). The effective theory of fluids at NLO and implications for dark energy. JCAP.

[35] G. Ballesteros, D. Comelli, L. Pilo, Thermodynamics of perfect fluids from scalar field theory. Phys. Rev. D **94**(2), 025034 (2016). 10.1103/PhysRevD.94.025034. arXiv:1605.05304 [hep-th]

[36] V. Faraoni, A. Giusti, Thermodynamics of scalar-tensor gravity. Phys. Rev. D **103**(12), L121501 (2021). 10.1103/PhysRevD.103.L121501. arXiv:2103.05389 [gr-qc]

[37] V. Faraoni, A. Giusti, A. Mentrelli, New approach to the thermodynamics of scalar-tensor gravity. Phys. Rev. D **104**(12), 124031 (2021). 10.1103/PhysRevD.104.124031. arXiv:2110.02368 [gr-qc]

[38] S. Giardino, V. Faraoni, A. Giusti, First-order thermodynamics of scalar-tensor cosmology. JCAP **04**, 053 (2022). 10.1088/1475-7516/2022/04/053. arXiv:2202.07393 [gr-qc]

[39] V. Faraoni, T. B. Françonnet, Stealth metastable state of scalar-tensor thermodynamics. Phys. Rev. D **105**(10), 104006 (2022). 10.1103/PhysRevD.105.104006. arXiv:2203.14934 [gr-qc]

[40] Wald RM (1984). General Relativity.

[41] A. Giusti, S. Giardino, V. Faraoni, Past-directed scalar field gradients and scalar-tensor thermodynamics. arXiv:2210.15348 [gr-qc] 10.1007/s10714-023-03095-7 PMC9995396 36911575

[42] Madsen MS (1988). Scalar Fields in Curved Space-times. Class. Quant. Grav..

[43] Pimentel LO (1989). Energy Momentum Tensor in the General Scalar-Tensor Theory. Class. Quant. Grav..

[44] V. Faraoni, J. Côté, Imperfect fluid description of modified gravities. Phys. Rev. D **98**(8), 084019 (2018). 10.1103/PhysRevD.98.084019. arXiv:1808.02427 [gr-qc]

[45] U. Nucamendi, R. De Arcia, T. Gonzalez, F. A. Horta-Rangel, I. Quiros, Equivalence between Horndeski and beyond Horndeski theories and imperfect fluids. Phys. Rev. D **102**(8), 084054 (2020). 10.1103/PhysRevD.102.084054. arXiv:1910.13026 [gr-qc]

[46] A. Giusti, S. Zentarra, L. Heisenberg, V. Faraoni, First-order thermodynamics of Horndeski gravity. Phys. Rev. D **105**(12), 124011 (2022). 10.1103/PhysRevD.105.124011. arXiv:2108.10706 [gr-qc]

[47] V. Faraoni, A. Giusti, S. Jose, S. Giardino, Peculiar thermal states in the first-order thermodynamics of gravity. Phys. Rev. D **106**(2), 024049 (2022). 10.1103/PhysRevD.106.024049. arXiv:2206.02046 [gr-qc]

[48] Eckart C (1940). The thermodynamics of irreversible processes. 3. Relativistic theory of the simple fluid. Phys. Rev..

[49] Landau LD, Lifshitz EM (1959). Fluid Mechanics.

[50] Nesseris S, Perivolaropoulos L (2007). Crossing the Phantom Divide: Theoretical Implications and Observational Status. JCAP.

[51] Di Valentino E, Melchiorri A, Silk J (2016). Reconciling Planck with the local value of $$H_0$$ in extended parameter space. Phys. Lett. B.

[52] E. Di Valentino, A. Mukherjee, A. A. Sen, Dark Energy with Phantom Crossing and the $$H_0$$ Tension. Entropy **23**(4), 404 (2021). 10.3390/e23040404. arXiv:2005.12587 [astro-ph.CO] 10.3390/e23040404 PMC8065785 33805393

[53] N. Aghanim et al. [Planck], Planck 2018 results. VI. Cosmological parameters. Astron. Astrophys. **641**, A6 (2020) [erratum: Astron. Astrophys. **652**, C4 (2021)]. 10.1051/0004-6361/201833910. arXiv:1807.06209 [astro-ph.CO]

[54] Misner CV, Thorne KS, Wheeler JA (1973). Gravitation.

[55] Kremer GM (2014). Diffusion of relativistic gas mixtures in gravitational fields. Phys. A.

[56] Chandrasekhar S (1943). Stochastic problems in physics and astronomy. Rev. Mod. Phys..

[57] Hu Y, Carruthers TF, Menyuk CR, Hutchinson MN, Urick VJ, Williams KJ (2015). Simulation of a partially depleted absorber (PDA) photodetector. Opt. Express.

[58] G. F. R. Ellis, Relativistic cosmology. Proc. Int. Sch. Phys. Fermi **47**, 104–182 (1971). Reprinted in Gen. Relativ. Gravit. **41**, 581–660 (2009). 10.1007/s10714-009-0760-7

[59] Nojiri S, Odintsov SD (2004). The Final state and thermodynamics of dark energy universe. Phys. Rev. D.

[60] Gonzalez-Diaz PF, Siguenza CL (2004). Phantom thermodynamics. Nucl. Phys. B.

[61] Gonzalez-Diaz PF, Siguenza CL (2004). The fate of black holes in an accelerating universe. Phys. Lett. B.

[62] Lima JAS, Alcaniz JS (2004). Thermodynamics and spectral distribution of dark energy. Phys. Lett. B.

[63] E. Babichev, V. Dokuchaev, Y. Eroshenko, Black hole mass decreasing due to phantom energy accretion. arXiv:gr-qc/0507119 [gr-qc] 10.1103/PhysRevLett.93.021102 15323894

[64] Izquierdo G, Pavon D (2006). Dark energy and the generalized second law. Phys. Lett. B.

[65] G. Izquierdo, D. Pavon, The Generalized second law in dark energy dominated universes. 10.1142/9789812834300_0249. arXiv:gr-qc/0612092 [gr-qc]

[66] Setare MR (2006). Generalized second law of thermodynamics in quintom dominated universe. Phys. Lett. B.

[67] W. Buchmuller, J. Jaeckel, Entropy Growth and the Dark Energy Equation of State. arXiv:astro-ph/0610835 [astro-ph]

[68] Gong Y, Wang B, Wang A (2007). On thermodynamical properties of dark energy. Phys. Rev. D.

[69] Gong Y, Wang B, Wang A (2007). Thermodynamical properties of the Universe with dark energy. JCAP.

[70] J. A. de Freitas Pacheco, J. E. Horvath, Generalized second law and phantom cosmology: Accreting black holes. Class. Quant. Grav. **24**, 5427–5434 (2007). 10.1088/0264-9381/24/22/007. arXiv:0709.1240 [gr-qc]

[71] Pereira SH, Lima JAS (2008). On Phantom Thermodynamics. Phys. Lett. B.

[72] Klemm D, Vanzo L (2004). Aspects of quantum gravity in de Sitter spaces. JCAP.

[73] Bekenstein JD (1974). Exact solutions of Einstein conformal scalar equations. Ann. Phys..

[74] Sotiriou TP, Faraoni V, Liberati S (2008). Int. J. Mod. Phys. D.

[75] I. Arsene et al., [BRAHMS], “Quark gluon plasma and color glass condensate at RHIC? The Perspective from the BRAHMS experiment,”. Nucl. Phys. A **757**, 1–27 (2005). 10.1016/j.nuclphysa.2005.02.130. arXiv:nucl-ex/0410020 [nucl-ex]

[76] Back BB (2005). [PHOBOS], “The PHOBOS perspective on discoveries at RHIC,”. Nucl. Phys. A.

[77] Adams J (2005). [STAR], “Experimental and theoretical challenges in the search for the quark gluon plasma: The STAR Collaboration’s critical assessment of the evidence from RHIC collisions”. Nucl. Phys. A.

[78] Adcox K (2005). [PHENIX], “Formation of dense partonic matter in relativistic nucleus-nucleus collisions at RHIC: Experimental evaluation by the PHENIX collaboration,”. Nucl. Phys. A.

[79] A. Monnai, Landau and Eckart frames for relativistic fluids in nuclear collisions. Phys. Rev. C **100**(1), 014901 (2019). 10.1103/PhysRevC.100.014901. arXiv:1904.11940 [nucl-th]

